# Phosphorylation Modification Characteristics of Liver Injury-Related Proteins in *Klebsiella pneumoniae* Liver Abscess

**DOI:** 10.3390/pathogens15070739

**Published:** 2026-07-14

**Authors:** Chao Yan, Xuanfeng Liu, Yujie Chen, An Su, Xue Ren, Tingting Zhang, Jing Yuan

**Affiliations:** Department of Bacteriology, Capital Center for Children’s Health, Capital Institute of Pediatrics, Capital Medical University, Beijing 100020, China; cip_yanchao@163.com (C.Y.); xuanfenglau@163.com (X.L.); yujie5376@163.com (Y.C.); suan2025@163.com (A.S.); rlxue0529@163.com (X.R.); zhangtingt@163.com (T.Z.)

**Keywords:** *Klebsiella pneumoniae*, liver abscess, phosphoproteomics, post-translational modification, liver injury

## Abstract

This study aimed to use quantitative phosphoproteomics to explore phosphorylation characteristics in *Klebsiella pneumoniae* (*Kpn*)-induced liver abscess (KPLA) formation and liver injury. The *Kpn* strain LA-*Kpn006* was phenotypically and genotypically characterized. A murine model of KPLA was established via intragastric inoculation. Phosphoproteomics and bioinformatic analyses were conducted to identify and quantify phosphosites and phosphoproteins. LA-*Kpn006* displayed a hypermucoviscous phenotype, serotype K1, ST23 genotype, and harbored six major virulence genes. Inoculation induced liver colonization and typical histopathological abscess lesions. We quantified 3017 phosphoproteins covering 12,798 phosphosites (dominated by serine phosphorylation); 1723 proteins were upregulated and 425 downregulated. Bioinformatic analyses revealed remodeling in metabolism, stress response, signal transduction, and cytoskeleton organization. Upregulated proteins converged on fatty acid elongation, inositol phosphate metabolism, and the tricarboxylic acid cycle; downregulated proteins were enriched in PI3K–Akt, IL-17 signaling, and T-cell differentiation. Protein–protein interaction network analysis identified 10 key proteins (Src, Rac1, Actb, Hsp90aa1, Hsp90ab1, Egfr, Rps6, Pik3CA, Itgb1, and Fyn) that mediate inflammatory signaling, cytoskeleton remodeling, and immune infiltration. Dysregulated phosphorylation networks drive pathological metabolic adaptation, suppressed immune homeostasis, and cytoskeletal disorganization, collectively facilitating KPLA progression. The identified hub proteins and pathways represent high-value mechanistic targets and candidate therapeutic vulnerabilities for KPLA.

## 1. Introduction

*Klebsiella pneumoniae* (*Kpn*) is a major Gram-negative pathogen responsible for community-acquired and nosocomial infections [1]. Pyogenic liver abscess caused by *Kpn* (KPLA) is a potentially life-threatening abdominal infectious disease with significant mortality (approximately 5%), which was first described in Taiwan [2]. Since then, KPLA has been reported in parts of East Asia, including China, the Republic of Korea, and Singapore, accounting for 80% of all cases [3,4,5]. Subsequently, there have also been numerous related reports in the USA, Europe, and Australia [6,7]. Up to now, KPLA has surpassed liver abscess caused by *Escherichia coli* to become the predominant phenotype in the clinic.

Compared with classical *Kpn*, which is significantly correlated with pneumonia, studies have shown that KPLA is primarily caused by hypervirulent *Kpn* (hvKp) [8,9]. Notably, hvKp strains belonging to capsular serotype K1 and sequence type ST23 are epidemiologically predominant worldwide, and often cause infection in healthy individuals [10,11,12]. In addition, hvKp can carry various virulence genes related to the biosynthesis of capsular polysaccharides and lipopolysaccharide, the production of siderophores, as well as fimbriae for promoting bacterial replication, overcoming innate host immunity and maintaining infection, which contribute to playing important roles in the development and progression of liver abscesses [13,14]. However, the underlying precise molecular mechanism(s) detailing how *Kpn* causes liver abscess formation remain poorly understood.

Increasing evidence indicates that intestinal colonization and subsequent translocation represent a critical route for hvKp targeting the liver [15]. However, the host signaling cascades and post-translational regulatory networks that mediate hepatocellular injury, inflammatory dysregulation, and abscess formation remain poorly defined. Protein phosphorylation is a reversible and tightly controlled post-translational modification that dynamically modulates protein function, subcellular localization, complex assembly, and signal regulation [16,17]. In infectious diseases, pathogen-induced phosphorylation rewires host immunity, metabolism, and cell fate to favor colonization, inflammation, and tissue damage [18,19]. Numerous studies indicate that protein phosphorylation exerts a critical regulatory role in the pathogenesis and progression of liver injury [20,21,22]. Despite its centrality to host–pathogen interplay, a comprehensive phosphoproteomic landscape of KPLA-associated liver injury has not been established.

In this study, we established a murine KPLA model via intragastric hvKp administration and performed high-throughput quantitative phosphoproteomics. We mapped dysregulated phosphoproteins, enriched functional modules, and core signaling pathways, aiming to reveal the phosphorylation-dependent mechanisms driving hvKp-induced liver pathogenesis and identify potential intervention targets.

## 2. Materials and Methods

### 2.1. Bacterial Strain and Culture

The hypervirulent *Klebsiella pneumoniae* strain LA-*Kpn006* was isolated from the aspirate of a patient with pyogenic liver abscess. Species identification was confirmed using an automated microbial identification system (MALDI-TOF MS, bioMérieux, Marcy-l’Étoile, France), and confirmed using 16S rRNA sequencing. Bacteria were cultured in a yeast extract (Oxoid, Basingstoke, UK), peptone (Oxoid, Basingstoke, UK) and dextrose (Solarbio, Beijing, China) medium at 37 °C with shaking at 180 rpm. Then, 2 mL of the quantified bacterial suspension was centrifuged at 12,000 rpm for 5 min (Thermo Fisher Scientific, Waltham, MA, USA), and the supernatant was discarded. The bacterial pellet was washed three times with sterile PBS (HyClone, Logan, UT, USA) and resuspended in 1 mL of sterile PBS with thorough mixing by vortexing (Thermo Fisher Scientific, Waltham, MA, USA). The bacterial suspension was diluted to a final concentration of 2 × 10^9^ CFU/mL based on the counting results. To ensure bacterial viability and improve the success rate of model establishment, all bacterial suspensions were freshly prepared immediately before intragastric administration.

### 2.2. Strain Characterization

The string test was used to assess hypermucoviscosity. Genomic DNA was extracted using a commercial DNeasy kit (Qiagen, Hilden, Germany) following the manufacturer’s instructions, and PCR was performed to determine capsular serotype (K1/K2/K5/K20/K54/K57) and virulence gene carriage (rmpA, rmpA2, allS, iroN, iutA, mrkD) [10,22,23]. MLST analysis was performed as described on the Pasteur Institute MLST website (https://bigsdb.pasteur.fr/klebsiella/, accessed on 13 May 2026).

### 2.3. Murine KPLA Model

Specific pathogen-free C57BL/6 mice (6–8 weeks, 20–22 g) were purchased from Charles River Laboratories (Beijing, China). All experiments were approved by the Ethics Committee of Capital Institute of Pediatrics (No. DWLL2025010) and conducted in compliance with the 3R principles and institutional animal welfare guidelines. Mice were randomized into control and infected groups (*n* = 3 per group). After an 8 h fast, mice received 200 μL saturated sodium bicarbonate (Macklin, Shanghai, China) to neutralize gastric acid, followed by intragastric inoculation of 4 × 10^8^ CFU mid-log-phase LA-*Kpn006* [24]. The mice were fasted for an additional 2 h and then given free access to food and water. Then they were euthanized at 3 days post-infection for tissue and serum collection. Three biological replicates per group are widely adopted for exploratory quantitative phosphoproteomics in pathogen-infected mouse models. All samples were processed in parallel with unified extraction, enrichment and labeling workflows to minimize technical variation.

### 2.4. Liver Function and Histopathology

Serum alanine transaminase (ALT) and aspartate transaminase (AST) were measured using commercial kits (Nanjing Jiancheng Bioengineering Institute, Nanjing, China). Liver tissues were fixed in 10% neutral formalin (Solarbio, Beijing, China), paraffin-embedded, sectioned, and stained with hematoxylin and eosin (H&E) for histopathological examination.

### 2.5. Phosphoproteomic Analysis

Liver tissues were lysed in ice-cold RIPA buffer (Solarbio, Beijing, China), proteins were extracted, digested with trypsin (Promega, Madison, WI, USA), and phosphopeptides were enriched using immobilized metal affinity chromatography. All samples were processed individually without pre-pooling. All six samples were analyzed in one batch to minimize batch effects. Peptides were analyzed by LC–MS/MS in data-independent acquisition (DIA) mode on an Orbitrap Exploris 480 system. Spectronaut (v18) was used for database searching and label-free quantification; false discovery rate was set to 1% for proteins, peptides, and PSMs. A phosphosite localization probability threshold of ≥0.75 was applied for high-confidence site filtering. Standard search parameters were applied: trypsin/P digestion with up to two missed cleavages, variable modifications of phosphorylation and oxidation, 10 ppm precursor tolerance and 0.02 Da fragment tolerance.

For data preprocessing, all quantitative values underwent log_2_ transformation to meet the assumptions of parametric tests. The relative abundance of each phosphorylation site was normalized to its corresponding parent protein level to eliminate the confounding effect of total protein expression. Missing values were imputed via the k-nearest neighbors (KNN) algorithm. 

For differential analysis, Levene’s test was first performed to assess variance homogeneity between groups. Welch’s *t*-test was applied for groups with unequal variance, and Student’s *t*-test for groups with equal variance. Differentially phosphorylated proteins were defined as |fold change| ≥ 1.5 and *p* < 0.05. This threshold was set for exploratory discovery.

### 2.6. Bioinformatics and Statistics

Gene Ontology (GO), Kyoto Encyclopedia of Genes and Genomes (KEGG) pathway, and protein domain enrichment analyses were performed using standard pipelines. Protein–protein interaction (PPI) networks were constructed using STRING (v11.5) (confidence score > 0.7) and visualized with visNetwork (v2.1.2). Raw phosphopeptide intensity data were log2-transformed, followed by median centering normalization for variance stabilization. Statistical analyses were performed using SPSS 30.0. Fisher’s exact test and χ^2^ test were applied as appropriate. *p* < 0.05 was considered statistically significant. Benjamini–Hochberg FDR correction was performed as a stringent reference.

## 3. Results

### 3.1. Phenotypic and Genotypic Features of Strain LA-Kpn006

**Figure 1:**
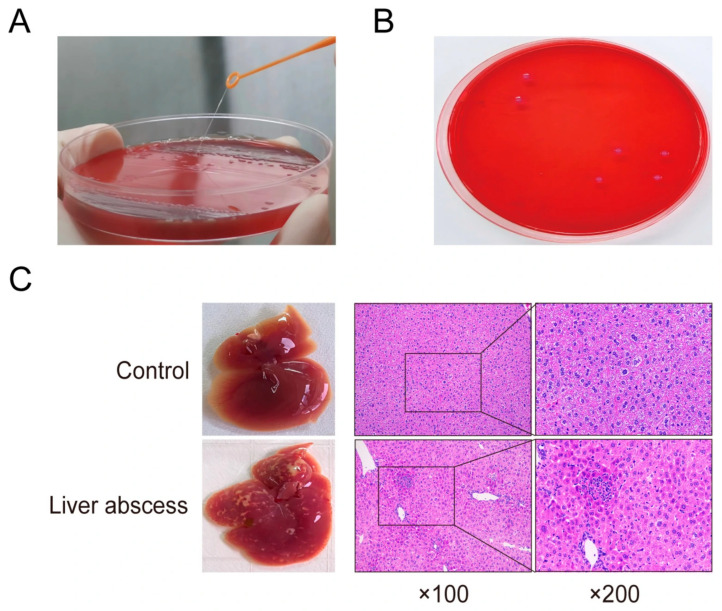
Phenotypic characteristics of LA-*Kpn006* and pathological changes in liver tissues of KPLA mice. (**A**) String test showing hypermucoviscous phenotype of strain LA-*Kpn006*; (**B**) Bacterial colonization in liver homogenates of KPLA model mice; (**C**) Histopathological changes (H&E staining) in liver tissues from control and KPLA mice.

LA-*Kpn006* exhibited a positive string test (mucus strand length > 10 mm), which is a typical phenotype of hypervirulent *Klebsiella pneumoniae* (Figure 1A). Molecular characterization further confirmed that this strain belonged to capsular serotype K1 and multilocus sequence type ST23, the two most clinically dominant and epidemiologically prevalent genotypes associated with severe invasive infections. Moreover, PCR detection verified that LA-*Kpn006* harbored all six virulence genes known to be closely associated with the high pathogenicity, hyperinvasiveness, and enhanced virulence of hvKp strains.

### 3.2. Establishment of the KPLA Model

At 3 days post-inoculation, viable hvKp was successfully detected in liver homogenates, with a bacterial load of approximately 1 × 10^3^ CFU/g, confirming stable colonization of the liver by the strain (Figure 1B). Meanwhile, serum levels of ALT and AST, key indicators of hepatocellular injury, were significantly increased in the infected group compared with the control group (*p* < 0.05), indicating severe liver damage induced by hvKp infection. Gross morphological examination showed obvious hepatomegaly and typical macroscopic abscess lesions on the liver surface. Histological examination via H&E staining further demonstrated complete disruption of the normal hepatic lobule structure, extensive hepatocyte necrosis, massive inflammatory cell infiltration, and distinct abscess formation in the liver tissue, which were consistent with the typical pathological features of KPLA (Figure 1C).

### 3.3. Phosphoproteomic Profiling

**Figure 2:**
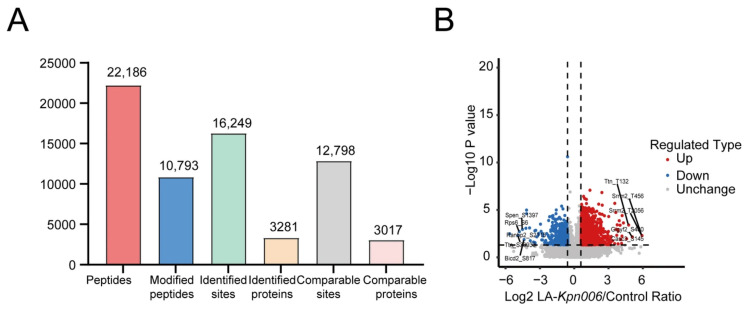
Identification and quantitative distribution of phosphorylation sites and proteins in mouse liver tissues. (**A**) Numbers of identified and quantifiable phosphorylation sites and phosphoproteins; (**B**) Volcano plot showing significantly upregulated and downregulated phosphorylation sites in KPLA liver tissues.

The results showed that a total of 16,249 phosphorylation sites and 3281 phosphorylated proteins were identified, among which 12,798 sites and 3017 proteins were quantified (Figure 2A). Among all phosphorylation sites, 13,921 (85.68%) were serine (Ser, S) residues, 1966 (12.10%) were threonine (Thr, T) residues, and 362 (2.22%) were tyrosine (Tyr, Y) residues. Differentially expressed phosphorylated proteins were screened by fold change and *t*-test. A *p* value < 0.05 and a fold change > 1.5 were set as the thresholds for significant upregulation, while a fold change < 1/1.5 was set as the threshold for significant downregulation. According to the above criteria, 1723 phosphorylated proteins (3176 sites) were significantly upregulated, and 425 phosphorylated proteins (491 sites) were significantly downregulated in the liver tissues of the KPLA mouse model (Figure 2B).

### 3.4. Gene Ontology Annotation of Dysregulated Phosphoproteins

**Figure 3:**
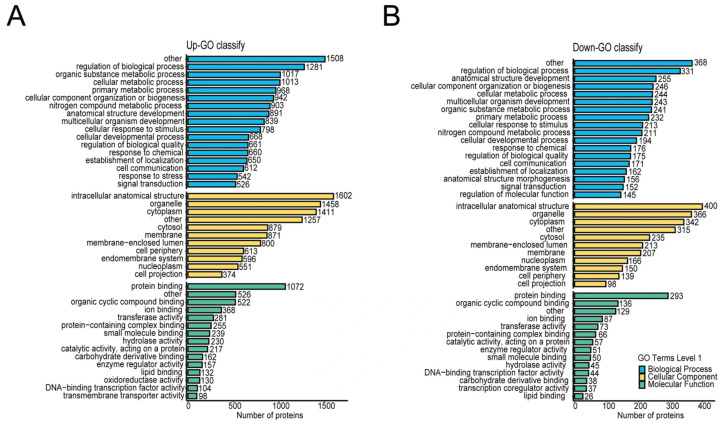
GO enrichment analysis of differentially phosphorylated proteins in KPLA mouse liver tissues. Distribution of differentially phosphorylated proteins in cellular components, molecular function, and biological process categories. (**A**) Upregulated phosphoproteins; (**B**) Downregulated phosphoproteins.

Based on the phosphoproteomic sequencing data, GO annotation was performed on 2148 differentially expressed phosphorylated proteins. As shown in Figure 3, for cellular components, the differentially phosphorylated proteins were mainly enriched in classic cellular compartments, including intracellular organelles, cytoplasm, cytosol, membrane structures, endomembrane system, nucleoplasm, and cell protrusions, suggesting that *Kpn* infection triggers global remodeling of protein phosphorylation in diverse subcellular structures of liver tissue, extensively affecting membrane functions, cytoplasmic signaling, and intranuclear biological processes. In terms of molecular functions, the differentially phosphorylated proteins were widely involved in protein–protein interaction, small molecule and ion binding, catalytic activity, transcriptional regulation, lipid and carbohydrate derivative binding, transmembrane transport, and other functions. Most core molecular functions were significantly enriched in both up- and downregulated proteins, indicating a global alteration of the molecular functional network in liver tissue after infection. Regarding biological processes, the differentially phosphorylated proteins participated in the regulation of biological processes, metabolic processes, cellular component organization, developmental processes, response to external stimuli, and signal transduction. Among them, downregulated phosphorylated proteins were specifically involved in anatomical structure morphogenesis and molecular function regulation, whereas upregulated phosphorylated proteins were significantly enriched in stress response-related biological processes, indicating functional differentiation mediated by distinct phosphorylation patterns in the KPLA model.

### 3.5. KEGG Enrichment of Dysregulated Phosphoproteins

**Figure 4:**
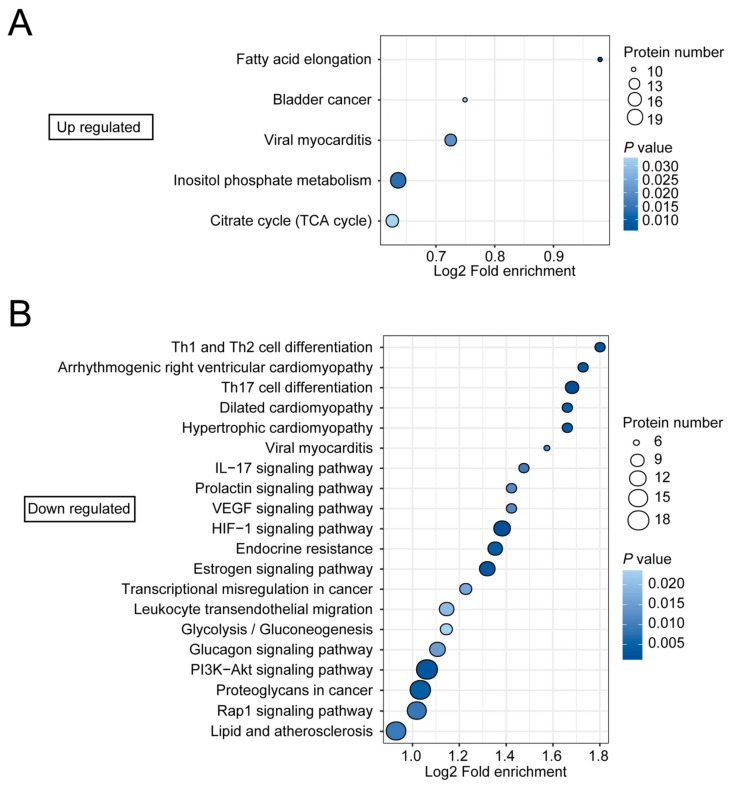
KEGG pathway enrichment analysis of differentially phosphorylated proteins. (**A**) Upregulated pathways; (**B**) Downregulated pathways.

KEGG pathway analysis was performed on the differentially phosphorylated proteins in KPLA liver tissues. The results revealed distinct regulatory differences in signaling pathways between up- and down-regulated phosphorylated proteins. Up-regulated phosphorylated proteins were mainly enriched in lipid and energy metabolism-related pathways, including fatty acid elongation, inositol phosphate metabolism, and the tricarboxylic acid cycle (Figure 4A). In contrast, down-regulated phosphorylated proteins were predominantly concentrated in immune and inflammatory pathways such as Th1/Th2 and Th17 cell differentiation, IL-17 signaling, and leukocyte transendothelial migration. In addition, pathways including phosphatidylinositol-3-kinase/protein kinase B (PI3K/Akt), Ras-related protein 1 (Rap1), Hypoxia-inducible factor 1 (HIF-1), Vascular endothelial growth factor (VEGF), glycolipid metabolism, and endocrine regulation were also significantly down-regulated (Figure 4B). These findings suggest that differentially phosphorylated proteins participate in liver injury induced by KPLA mainly through pathways related to lipid metabolism, energy metabolism, inositol signaling, immune inflammation, and cell survival and differentiation.

### 3.6. Protein Domain Enrichment of Dysregulated Phosphoproteins

Protein domain enrichment analysis demonstrated that upregulated domains were mainly enriched in Post-synaptic density protein 95, Drosophila disk large tumor suppressor 1, Zonula occludens-1 protein (PDZ), Variant Src Homology 3 domain (SH3), Filamin/Actin-Binding Protein 280 (ABP280), deleted Differentially Expressed in Normal and Neoplastic (dDENN), Sterile Alpha Motif (SAM), 4.1-Ezrin-Radixin-Moesin (FERM) family, Pleckstrin Homology (PH), and Ras Guanine Nucleotide Exchange Factor (RasGEF) domains, which are involved in cytoskeleton remodeling, protein–protein interaction, G protein signaling, membrane trafficking, and inflammatory transcriptional regulation (Table 1). Downregulated domains were dominated by Phosphotyrosine Binding (PTB), SH2, Chromo, AT-Rich Interaction Domain (ARID)/Binding of Retinoblastoma protein, Inhibitor of cell proliferation, and Global transcription regulator (BRIGHT), Phosphatase and Tensin Homolog (PTEN)-C2, Sec16 vesicle trafficking, Signal Transducer and Activator of Transcription (STAT) function, and zinc finger transcription domains, which are mainly implicated in tyrosine phosphorylation signaling, chromatin transcriptional regulation, vesicular transport, tumor suppression, and inflammatory homeostasis (Table 1). Notably, PDZ and FERM central domains were significantly enriched in both upregulated and downregulated proteins, suggesting that they may participate in the pathological processes of inflammatory activation, hepatocyte injury, and tissue homeostasis imbalance through bidirectional phosphorylation modification [25].

### 3.7. Core Proteins in the PPI Network

**Figure 5:**
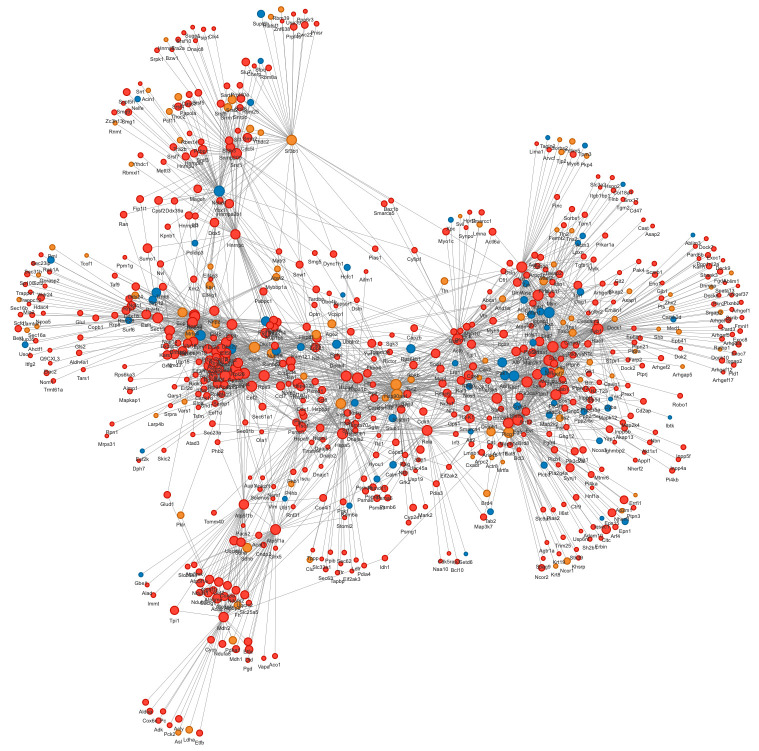
Protein–protein interaction network of differentially phosphorylated proteins. Nodes represent phosphorylated proteins; red = upregulated, blue = downregulated, yellow = bidirectionally regulated. Circle size indicates node degree. The interactive HTML file and raw node/link text files for this network are provided in Supplementary Materials for detailed browsing and analysis.

The top 50 phosphorylated proteins with the closest interactive relationships were selected to map the PPI network. The full set of 2148 differentially phosphorylated proteins was used as the input for STRING network construction. The final network includes both the input differential proteins and their predicted interacting partners. The “top 50” proteins are ranked by node degree from the completed network as the core subset for hub analysis. The results showed that the network consisted of 1593 nodes and 8573 edges, and most nodes possessed more than two interactive relationships (Figure 5). The top 10 proteins ranked by node degree were identified as core proteins (Table 2). Among them, Proto-oncogene tyrosine-protein kinase Src, Ras-related C3 botulinum toxin substrate 1 (Rac1), Beta-actin (Actb), Epidermal growth factor receptor (EGFR), Phosphatidylinositol-4,5-bisphosphate 3-kinase catalytic subunit alpha (Pik3CA), integrin gene (Itgb1) and Tyrosine-protein kinase Fyn were significantly upregulated; Heat shock protein (Hsp90) aa1, Hsp90ab1 and Ribosomal protein S6 (Rps6) exhibited bidirectional regulatory patterns. The node degree of these proteins ranged from 62 to 108. Collectively, these proteins possess high connectivity and serve as central regulators within the phosphorylation-mediated regulatory network of liver injury in KPLA.

## 4. Discussion

This study presents the first in-depth phosphoproteomic landscape of hvKp-induced liver abscess, revealing widespread phosphorylation-dependent remodeling of host metabolism, immunity, and cytoskeletal architecture. Our data establish that protein phosphorylation is a central regulatory pattern in KPLA pathogenesis and a key point for mechanistic dissection and therapeutic development.

The experimental strain exhibited a positive string test, with capsular serotype K1 and MLST ST23, and carried six major virulence genes, including *rmpA*, *rmpA2*, *allS*, *iroN*, *iutA*, and *mrkD*. These characteristics are typical of hypervirulent *Klebsiella pneumoniae* and are closely associated with high pathogenicity, hypermucoviscosity, iron acquisition, and adhesion ability [22,23,26]. Such genotypic and phenotypic features enable the strain to efficiently colonize, invade, and induce severe inflammatory damage in the liver, thereby contributing to the formation and progression of liver abscess.

Furthermore, we successfully established a murine model of KPLA via intragastric administration. Typical pathological changes were clearly observed in infected liver tissues, including structural destruction of hepatic lobules, massive inflammatory cell infiltration, hepatocellular necrosis, and obvious abscess formation. These results confirmed that the model was reliable and suitable for exploring the molecular mechanisms underlying hvKp-induced liver injury and pathological progression [24].

Notably, the culturable bacterial load is relatively low at day 3. Four factors explain this discrepancy. First, some bacteria enter a viable non-culturable state. Plate CFU underestimates the total bacterial burden. Second, liver abscesses distribute unevenly in the tissue. Single-site sampling may miss high-burden lesions. Third, innate immunity clears part of the culturable bacteria. Tissue damage persists longer than live bacteria. Fourth, our strain is a hypervirulent *K. pneumoniae*, which causes severe damage at low bacterial loads, and its virulence factors drive strong inflammatory responses. In addition, intragastric inoculation crosses the gut barrier, which filters most inoculated bacteria and naturally lowers liver colonization levels.

Phosphoproteomic quantification revealed 3667 dysregulated phosphosites in total, among which 3176 were significantly upregulated, and 491 were downregulated, demonstrating that hvKp disrupts host phosphorylation signaling to drive pathological progression. The predominance of serine phosphorylation implies that phosphorylation preferentially remodels the backbone conformational characteristics of serine over threonine residues during hvKp-induced liver injury [27].

Functional enrichment of phosphorylated proteins indicated that infection triggers a pro-inflammatory metabolic change; upregulated pathways strengthen lipid and energy metabolism to maintain inflammatory signaling and oxidative stress, while downregulated pathways suppress immune response, cell survival, and tissue repair. These changes exacerbate an inflammatory response, hepatic tissue injury, and subsequent abscess development. Notably, repression of PI3K–Akt and IL-17 signaling suggests impaired adaptive immunity and compromised tissue homeostasis, which may explain the persistence of infection and severity of liver injury. Mechanistically, the PI3K–Akt axis is central to regulating the development, differentiation, metabolic reprogramming, and effector functions of both myeloid and lymphoid immune cells, including macrophages, dendritic cells, and neutrophils, while the lymphoid lineage encompasses T cells, B cells, NK cells, and multiple innate lymphoid cell subsets, as well as innate-like lymphocytes, such as NKT cells and MAIT cells [28]. In parallel, IL-17 signaling is essential for host defense against extracellular bacteria and for maintaining hepatic immune tolerance; its inhibition disrupts local inflammatory control and tissue repair circuits, favoring persistent bacterial colonization and progressive parenchymal damage [29]. Consistent with our findings, prior studies have demonstrated that PI3K–Akt repression exacerbates liver injury by dysregulating macrophage polarization and impairing hepatocyte survival. As a key bioactive component of Scutellaria baicalensis Georgi, wogonin ameliorates hepatic damage and acetaminophen-related hepatotoxicity in mice by suppressing macrophage inflammatory activation via inhibiting the PI3K/AKT signaling pathway [30]. While IL-17 pathway impairment is closely linked to uncontrolled inflammatory disorders and increased host susceptibility to bacterial infection [31]. Collectively, these data support that co-suppression of PI3K–Akt and IL-17 signaling acts as a key molecular driver underlying ineffective adaptive immunity, disrupted hepatic homeostasis, and the severe, persistent infection observed in this KPLA model. This apparent discrepancy can be explained from three perspectives. First, bulk liver tissue is dominated by hepatocytes. The detected phosphorylation signals mainly reflect hepatocyte status rather than infiltrating immune cells, with a low proportion. Second, the downregulated pathways belong to adaptive immunity. The acute inflammatory lesions at day 3 are driven by innate immune cells. These two patterns act on separate immune branches. Third, pathway downregulation may represent host negative feedback. It is triggered by strong acute inflammation to avoid excessive tissue damage.

Protein domain analysis highlighted structural modules that govern protein–protein interaction, cytoskeleton dynamics, and transcriptional control. The bidirectional enrichment of PDZ and FERM domains implies context-dependent phosphorylation switches that coordinate inflammatory activation and cellular disorganization [32,33,34].

PPI network analysis prioritized 10 high-value hub proteins that integrate upstream signals and regulate downstream effector functions. Src, Rac1, Pik3CA, and EGFR control kinase signaling, cytoskeleton rearrangement, and immune cell infiltration [35,36,37,38]; Actb and Itgb1 mediate cellular structural integrity [39,40]; Hsp90 paralogs and Rps6 govern protein homeostasis and translation [41,42]. Among them, as an E3 ubiquitin ligase, NEDD4L negatively modulates PIK3CA protein abundance through ubiquitination and is essential for maintaining PI3K-AKT signaling homeostasis [37]. EGFR–HSP90 complex synergistically mediates acquired resistance to EGFR tyrosine kinase inhibitors and underpins the pathogenesis of EGFR-addicted tumors [38]. These hubs likely function as master regulators of KPLA pathogenesis and represent ideal candidates for targeted inhibition.

Several limitations should be noted: the sample size is modest, a single time point was analyzed, and in vivo functional validation is lacking. Future studies will expand animal cohorts, profile phosphorylation dynamics over time, and use genetic and pharmacological approaches to validate hub protein function.

## 5. Conclusions

Hypervirulent *Klebsiella pneumoniae* infection elicits extensive and functionally coordinated phosphoproteomic remodeling in mouse liver tissue. Dysregulated phosphorylation pathways drive metabolic reprogramming, immune dysregulation, and cytoskeletal disruption, collectively promoting liver abscess and tissue injury. The identified core proteins and signaling modules illuminate the molecular basis of KPLA and provide promising targets for novel diagnostic and therapeutic strategies against severe hvKp infection.

## Figures and Tables

**Table 1:** Protein domain enrichment analysis of differentially phosphorylated proteins.

Regulated Type	Domain Description	Mapping	*p* Value *
Up-regulated	PDZ domain	32	0.000
	Variant SH3 domain	19	0.001
	Filamin/ABP280 repeat	6	0.006
	dDENN domain	6	0.006
	SAM domain (Sterile alpha motif)	10	0.006
	FERM N-terminal domain	11	0.011
	FERM C-terminal PH-like domain	11	0.011
	uDENN domain	7	0.012
	RasGEF N-terminal motif	5	0.013
	TFIIS helical bundle-like domain	5	0.013
	PH domain	5	0.013
	FERM central domain	15	0.017
	SAB domain	4	0.032
	LisH	4	0.032
Down-regulated	Phosphotyrosine-binding domain	4	0.001
	SPOC domain	3	0.001
	Chromo (CHRromatin Organisation MOdifier) domain	4	0.008
	SH2 domain	9	0.009
	ARID/BRIGHT DNA-binding domain	3	0.012
	Villin headpiece domain	3	0.012
	C2 domain of PTEN tumour-suppressor protein	3	0.012
	Lyase	2	0.013
	Transferrin	2	0.013
	Vesicle coat trafficking protein Sec16 mid-region	2	0.013
	PDZ domain	11	0.017
	START domain	3	0.022
	PAS domain	3	0.022
	FERM central domain	6	0.031
	STAT protein, DNA-binding domain	2	0.035
	STAT protein, protein interaction domain	2	0.035
	Nuclear receptor coactivator	2	0.035
	Steroid receptor coactivator	2	0.035
	Sec23-binding domain of Sec16	2	0.035
	Zinc-fingers and homeoboxes C2H2 finger domain	2	0.035

* Fisher’s exact test *p* value.

**Table 2:** Top 10 hub proteins in the PPI network of differentially phosphorylated proteins.

Protein Description (Gene Name)	Degree	Position *	Regulated Type
Proto-oncogene tyrosine-protein kinase Src	108	36	Up
Ras-related C3 botulinum toxin substrate 1 (*Rac1*)	99	108	Up
β-Actin (*Actb*)	96	106	Up
Heat shock protein HSP 90-alpha (*Hsp90aa1*)	83	316	All
Heat shock protein HSP 90-beta (*Hsp90ab1*)	80	460	All
Epidermal growth factor receptor (*Egfr*)	69	1197	Up
Small ribosomal subunit protein eS6 (*Rps6*)	69	242	All
Phosphatidylinositol 4,5-bisphosphate 3-kinase catalytic subunit alpha (*Pik3ca*)	64	1061	Up
Integrin beta-1 (*Itgb1*)	63	777	Up
Tyrosine-protein kinase Fyn	62	21	Up

* “Position” indicates the ranking of each protein sorted by absolute |log2 fold change| among all 2148 differentially phosphorylated proteins.

## Data Availability

The mass spectrometry proteomics data have been deposited in the OMIX repository of the China National Center for Bioinformation (CNCB), with the accession number OMIX018608.

## Supplementary Materials

The following supporting information can be downloaded at: https://www.mdpi.com/article/10.3390/pathogens15070739/s1, Interactive PPI network files: HTML visualization page, node text file, link text file and log file for detailed network browsing.

## Abbreviations

The following abbreviations are used in this manuscript:

ABP280	Filamin/Actin-Binding Protein 280
Actb	Beta-actin
ALT	Alanine transaminase
ARID	AT-Rich Interaction Domain
AST	Aspartate transaminase
BRIGHT	Binding of Retinoblastoma protein, Inhibitor of cell proliferation, and Global transcription regulator
CFU	Colony Forming Unit
dDENN	deleted Differentially Expressed in Normal and Neoplastic
Egfr	Epidermal growth factor receptor
FERM	4.1-Ezrin-Radixin-Moesin
GO	Gene Ontology
H&E	Hematoxylin and eosin
HIF-1	Hypoxia-inducible factor 1
Hsp90aa1	Heat shock protein HSP 90-alpha
Hsp90ab1	Heat shock protein HSP 90-beta
hvKp	Hypervirulent *Klebsiella pneumoniae*
Itgb1	Integrin beta-1
KEGG	Kyoto Encyclopedia of Genes and Genomes
*Kpn*	*Klebsiella pneumoniae*
KPLA	Klebsiella pneumoniae liver abscess
LC–MS/MS	Liquid chromatography with tandem mass spectrometry
MLST	Multilocus Sequence Typing
PBS	Phosphate-Buffered Saline
PDZ	Post-synaptic density protein 95, Drosophila disc large tumor suppressor 1, Zonula occludens-1 protein
PH	Pleckstrin Homology
PI3K/Akt	Phosphatidylinositol-3-kinase/protein kinase B
Pik3ca	Phosphatidylinositol 4,5-bisphosphate 3-kinase catalytic subunit alpha
PPI	Protein–protein interaction
PSMs	Peptide spectrum matches
PTB	Phosphotyrosine Binding
PTEN	Phosphatase and Tensin Homolog
Rac1	Ras-related C3 botulinum toxin substrate 1
Rap1	Ras-related protein 1
RasGEF	Ras Guanine Nucleotide Exchange Factor
Rps6	Ribosomal protein S6
SAM	Sterile Alpha Motif
SH2	Src Homology 2
SH3	Src Homology 3
SPF	Specific Pathogen-Free
STAT	Signal Transducer and Activator of Transcription
VEGF	Vascular endothelial growth factor
